# The HIV-1 Transgenic Rat: Relevance for HIV Noninfectious Comorbidity Research

**DOI:** 10.3390/microorganisms8111643

**Published:** 2020-10-23

**Authors:** Frank Denaro, Francesca Benedetti, Myla D. Worthington, Giovanni Scapagnini, Christopher C. Krauss, Sumiko Williams, Joseph Bryant, Harry Davis, Olga S. Latinovic, Davide Zella

**Affiliations:** 1 Department of Biology, Morgan State University, Baltimore, MD 21251, USA; Frank.Denaro@morgan.edu (F.D.); mywor1@morgan.edu (M.D.W.); chkra1@morgan.edu (C.C.K.); SumWilliams@ihv.umaryland.edu (S.W.); 2 Institute of Human Virology, School of Medicine, University of Maryland, Baltimore, MD 21201, USA; fbenedetti@ihv.umaryland.edu (F.B.); jbryant@ihv.umaryland.edu (J.B.); hdavis@ihv.umaryland.edu (H.D.); olatinovic@ihv.umaryland.edu (O.S.L.); 3 Department of Biochemistry and Molecular Biology, University of Maryland, Baltimore, MD 21201, USA; 4 Department of Medicine and Health Science, University of Molise, Via F. De Sanctis, 86100 Campobasso, Italy; giovanni.scapagnini@unimol.it; 5 Institute of Human Virology-Animal Core Division, School of Medicine, University of Maryland, Baltimore, MD 21201, USA; 6 Department of Microbiology and Immunology, University of Maryland, Baltimore, MD 21201, USA

**Keywords:** HIV-1, noninfectious comorbidities, selective cell vulnerability, neuronal degeneration, gp120, Tat, CXCR4, HIV-1 transgenic rat

## Abstract

HIV noninfectious comorbidities (NICMs) are a current healthcare challenge. The situation is further complicated as there are very few effective models that can be used for NICM research. Previous research has supported the use of the HIV-1 transgenic rat (HIV-1TGR) as a model for the study of HIV/AIDS. However, additional studies are needed to confirm whether this model has features that would support NICM research. A demonstration of the utility of the HIV-1TGR model would be to show that the HIV-1TGR has cellular receptors able to bind HIV proteins, as this would be relevant for the study of cell-specific tissue pathology. In fact, an increased frequency of HIV receptors on a specific cell type may increase tissue vulnerability since binding to HIV proteins would eventually result in cell dysfunction and death. Evidence suggests that observations of selective cellular vulnerability in this model are consistent with some specific tissue vulnerabilities seen in NICMs. We identified CXCR4-expressing cells in the brain, while specific markers for neuronal degeneration demonstrated that the same neural types were dying. We also confirm the presence of gp120 and Tat by immunocytochemistry in the spleen, as previously reported. However, we observed very rare positive cells in the brain. This underscores the point that gp120, which has been reported as detected in the sera and CSF, is a likely source to which these CXCR4-positive cells are exposed. This alternative appears more probable than the local production of gp120. Further studies may indicate some level of local production, but that will not eliminate the role of receptor-mediated pathology. The binding of gp120 to the CXCR4 receptor on neurons and other neural cell types in the HIV-1TGR can thus explain the phenomena of selective cell death. Selective cellular vulnerability may be a contributing factor to the development of NICMs. Our data indicate that the HIV-1TGR can be an effective model for the studies of HIV NICMs because of the difference in the regional expression of CXCR4 in rat tissues, thus leading to specific organ pathology. This also suggests that the model can be used in the development of therapeutic options.

## 1. Introduction

The disease process of human immunodeficiency virus (HIV) infection has evolved with the use of very effective antivirals. When patients are under treatment, the number and frequency of opportunistic infections are markedly decreased. The advances in antiviral therapy have resulted in the marked suppression of HIV replication. This has resulted in the almost complete suppression of HIV symptoms [1] and opportunistic infections [2]. However, over time, patients may display noninfectious comorbidities (NICMs) even with treatment [3]. Eventually, NICMs can increase in severity, affecting the brain, heart, kidney, eye, and immune system [4,5]. These NICMs are known to intersect with healthcare disparities, which further complicates their medical treatment [6].

Monocytes and macrophages are important components of the immune system. They have been shown to be one of the major targets of HIV. Infected monocyte/macrophage cells serve as viral reservoirs and vehicles of virus dissemination throughout the body. Since the cytopathic effect of HIV is minimal in these cell types, they are long-lived and can continue to release the virus [7]. Integrated provirus can escape detection by the immunologic surveillance system in macrophages, and its presence induces an impairment of numerous immunoregulatory activities [7,8,9,10,11]. Changes of cytokine expression levels in HIV-1-infected macrophages have been reported in several in-vitro and in-vivo studies. In particular, an overproduction of the proinflammatory cytokines TNF-α [10,12,13,14,15], IL-1β [10,12,13,14], IL-6 [14,15,16], and GM-CSF [17] has been shown. On the contrary, impaired production of the proinflammatory cytokine IL-12 by HIV-infected monocytes/macrophages has been identified [18,19,20,21]. Controversial data regarding anti-inflammatory cytokine IL-10 has been presented: while some authors reported an increased expression of this factor [22], no alterations have been identified by several other laboratories [18,23,24,25]. The expression of several other cytokines, such as IFN-α [26,27], IFN-β [28], M-CSF [29], GM-CSF [17], MIP-1α [30,31,32], and RANTES [33], has been demonstrated to be increased in HIV-infected macrophages. This cellular dysregulation can contribute to both acute and long-term immune dysregulation found in HIV infections. Some of these changes have been addressed with therapy, but immune dysregulation and HIV cell infection can continue throughout the patient’s lifetime, albeit at very low levels. 

One continuing effect produced by the macrophage/monocytes is a low level of HIV production or HIV protein production. Therefore, the macrophage/monocyte is a reservoir cell that can contribute to NICMs [34,35,36,37]. For this reason, NICMs are believed to result from incomplete HIV suppression in the reservoir cells [38]. In this regard, it has been proposed that the emergence of HIV NICMs is a function of the levels of HIV proteins produced by HIV reservoirs, together with the ensuing altered immune response. This is a long-term chronic process, with the low levels of viral proteins exerting their effect over time. HIV viral proteins, produced from the infected reservoir cells, are believed to be generated in sufficient quantities that can result in altered cell functions [39]. The persistence of reservoirs may eventually contribute to the development of diseases in the brain, heart, and kidney. In fact, these viral proteins can cause immune dysregulation, alter oxidative stress, and produce cell dysfunction that ultimately leads to cell death [40] and to NICMs.

In view of the changing characteristics of the disease, it is thus desirable to develop in vivo model systems to study these emerging types of pathology. However, most of the experimental models developed so far to study the effects of HIV infection have focused on the goal of eliminating the virus or were conceived for vaccine development studies. Conversely, understanding and reproducing NICMs requires additional model characteristics. One model system approach is to make transgenic animals, which would mimic in a more physiological way the chronic exposure to HIV proteins that have been implicated in NICM development. The first iteration of an animal system was the HIV transgenic mouse [41], which expressed low levels of HIV proteins, hardly inducible by external stimuli [42]. It soon became clear that higher levels or upregulation of the transgenic products may offer additional advantages by increasing cell dysfunction that can result in increased measurable pathology. 

We choose the rat as the species to develop a transgenic HIV model (HIV-1TGR) [43] due to the prominent ability of Tat to regulate the HIV viral promoter. The rat also has compatible cellular cofactors that permit the production of HIV viral proteins [44]. These transgene products can then be identified immunocytochemically in the HIV-1TGR [43], and gp120 can also be quantified in the sera and cerebral spinal fluid (CSF) [45].

Previous studies have also demonstrated that HIV-1TGR macrophages produce HIV transgenic products [46]. Moreover, contrary to the mouse model, the rat possesses a CXCR4 cell surface molecule that is highly homologous to its human counterpart. Additionally, gp120 was demonstrated to bind to the HIV-1TGR CXCR4 receptor [47,48]. 

To provide evidence that would support the use of this model in HIV NICM research, we first aimed at identifying the histological location of the HIV-1 receptor CXCR4 in the brain of the HIV-1TGR. Next, we sought to determine if CXCR4-positive cells also presented with pathology. There have been a number of studies demonstrating neuropathology in different locations, such as the cortex [49], the hippocampus [50], and the dopaminergic system [51]. 

The presence of CXCR4 receptors would provide an explanation for the specificity of the pathology observed in these cells. Regarding the source of gp120, one possible explanation is the local production of HIV-1TGR products. Another possible mechanism is that exposure occurs from sera or CSF [43,45].

These points can be addressed at the level of histological analysis: first, by confirming the presence of CXCR4 on cells by immunocytochemistry; then, by insights into whether the production of gp120 is local or from another source. This can be determined, in part, by immunocytochemistry of gp120 and Tat. To determine neuron-related pathology, a specific stain for neurodegeneration was used. This information would provide a starting point to address specific tissue or organ pathology. Analysis of questions regarding the development of strategies to prevent HIV-induced cell death and dysfunction are among the possibilities that this model can offer. In addition, by determining some of the pathways that can lead to cell death, pharmaceuticals can be tested.

## 2. Materials and Methods

### 2.1. Animals 

Nontransgenic age-matched control (F344/Hsd) rats and male HIV-1TGR (F344/Hsd) were used; an extensive description of the model and the HIV-transgene inserted in the rat genome can be found in [43]. Rats were housed in a temperature-controlled environment. The light/dark cycle was set for 12 h light. Food and water were provided ad lib. All studies were approved by the Institutional Animal Care and Use Committee, Office of Welfare Assurance, School of Medicine, University of Maryland, Baltimore (protocols number 0514005 and 0417004, 06/09/2014 expired 05/14/2017). The animals used in the study were all more than 1 year old. The animals were maintained in the Animal Models Core at the Institute of Human Virology.

### 2.2. Tissue Processing 

Tissue was processed in the Biomedical Research Core Facility at Morgan State University. For cupric silver stains (CSS), six HIV-1TGRs and six controls were used. These animals were deeply anesthetized and were cardiac perfused with neutral buffered saline, followed by buffered formalin. Coronal frozen sections were produced at 75 microns and stored in buffered formalin at 6 °C. Every 50th section was processed for silver degeneration, and the adjacent section for hematoxylin and eosin (H&E). The stereotaxic atlas for the rat brain [52] was used to verify anatomical structures and the anterior/posterior position of the coronal sections. Six HIV-1TGRs and six controls were also perfused, and the brain and spleen were processed for paraffin sections. Frozen and paraffin sections, including the forebrain, striatum, hippocampus, substantia nigra, cerebellum, and brain stem, were produced. Brains and spleens from four HIV-1TGRs and 4 controls were used for CXCR4 immunocytochemistry. The fresh brain and spleen tissue were embedded in cryo-embedding media (OCT) and frozen in cool isopentane with liquid nitrogen. Tissues were then cut on a cryostat into 10- to 20-micron sections and immediately fixed in anhydrous acetone, chilled to −50 °C. Sections were then finally stored at −80 °C. 

### 2.3. Immunocytochemistry for Transgene Products gp120 and Tat 

Immunocytochemistry of Tat and gp120 was performed as previously described [43]. Goat polyclonal anti gp120 antibody (Abcam, Cambridge, MA, USA) and rabbit Tat polyclonal antibody (ThermoFisher Scientific, Waltham, MA USA) were used.

### 2.4. Immunochemistry for CXCR4

Anti-CXCR4 antibody (Abcam, Cambridge, MA, USA) was used with the ABC method (Vectastain ABC HRP Kit PK-4000 – Vector Laboratories, Burlingame, CA, USA). The chromogen used was DAB (3,3′-diaminobenzidine). 

### 2.5. Special Stain 

The cupric silver degeneration stain was used, following the method previously described by de Olmo et al. [53,54]. Sections were counterstained with neutral red.

### 2.6. Routine Stain 

H&E staining was performed on samples of all tissue to evaluate if there were any noticeable pathologic changes. 

Matched-age transgenic rats were used as negative controls for both the silver stain and the immunocytochemistry to the HIV transgenic products.

### 2.7. Slide Review of H&E Serial Sections

Brains were placed in rat brain sectioning molds and sliced coronally at 5 mm intervals. These coronal sections were inspected for gross changes before processing. The spleen was also sectioned into 5 mm sections, and groups were either processed into formaldehyde-fixed or -unfixed cryostat frozen sections. The serial coronal sections of both the frozen (formalin-fixed) and paraffin sections were reviewed for pathologic changes. Areas observed included the cortex, the caudate/putamen, the hippocampus, and the brain stem. Micrographs documented any relevant changes.

### 2.8. Slide Review Silver Stain

Sections adjacent to the frozen H&E sections were processed with the cupric silver degeneration stain (CSS). Different degrees of degeneration can sometimes be ascertained by the degrees of granularity of silver impregnation. Particular attention was paid to those impregnated cells that maintained their morphology so that the neuronal cell type could be identified. Very granular impregnation suggests the neuron underwent degeneration at an earlier time point. Very darkly labeled cells suggest a more recent cell death. 

### 2.9. Slide Review of CXCR4 Immunostaining 

Frozen coronal sections throughout the brains were stained with CXCR4 and analyzed for cell type. We compared these cells to the identified degenerating neurons in the CSS formaldehyde-fixed tissue. In this way, it was possible to correlate the morphology of CXCR4-labeled cells/neurons and the silver stain for degenerating neurons. 

### 2.10. Imaging

Image collection took place in the Biomedical Research Core Facility at of Morgan State University and the Institute of Human Virology. An Olympus BX43 microscope (DP72 camera) was used for photomicroscopy. A Nikon Optiphot, with a 60× water immersion objective and through focus capability of 125 microns, was used to evaluate the silver degeneration stain. This allowed the detailed observation of neuronal morphology and processes. Images were digitally collected using CellSens Standard software (Olympus).

## 3. Results

Macroscopic examination of the brains revealed that they were grossly normal. No signs of hemorrhages, softening, necrosis, or swelling were identified. Microscopic examination of the H&E sections revealed subtle abnormalities to the neurons in the transgenic animals. These changes included alterations to nuclear chromatin. Previously, it has been demonstrated that apoptotic changes take place in cells of the nervous system, which include both neurons and endothelial cells [43]. In some cases, the neurons displayed the “dead and red” appearance. These changes were found through the brain but were infrequent. Interestingly, inflammatory infiltrates were not found around or associated with these dead or dying cells. The same was true when dead neurons were confirmed using CSS. The counterstain did not reveal any inflammation cells. The cortex, hippocampus, and caudate/putamen all displayed clear neuronal degeneration by the silver stain. The morphology of the impregnation cells permitted the neuronal cell type to be identified. Early-stage neuronal death typically leaves cells well-infiltrated with the silver label. However, neurons that have died at an earlier time point often display silver particulates. This is often the case with the neuronal processes. They may not be visible for some degenerating neurons. The frequency of labeled cells was low, so quantification was not undertaken. However, the labeled cells allowed morphological identification and correlation with the CXCR4-labeled cell types.

### 3.1. Cortical Pathology 

**Figure 1:**
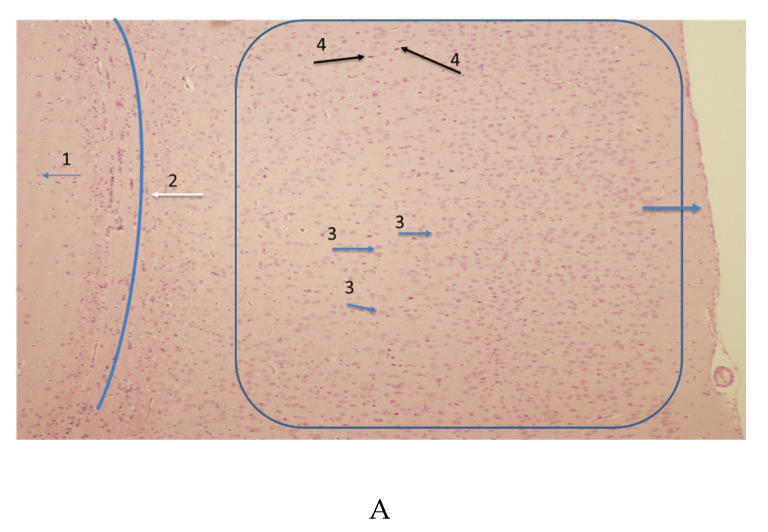

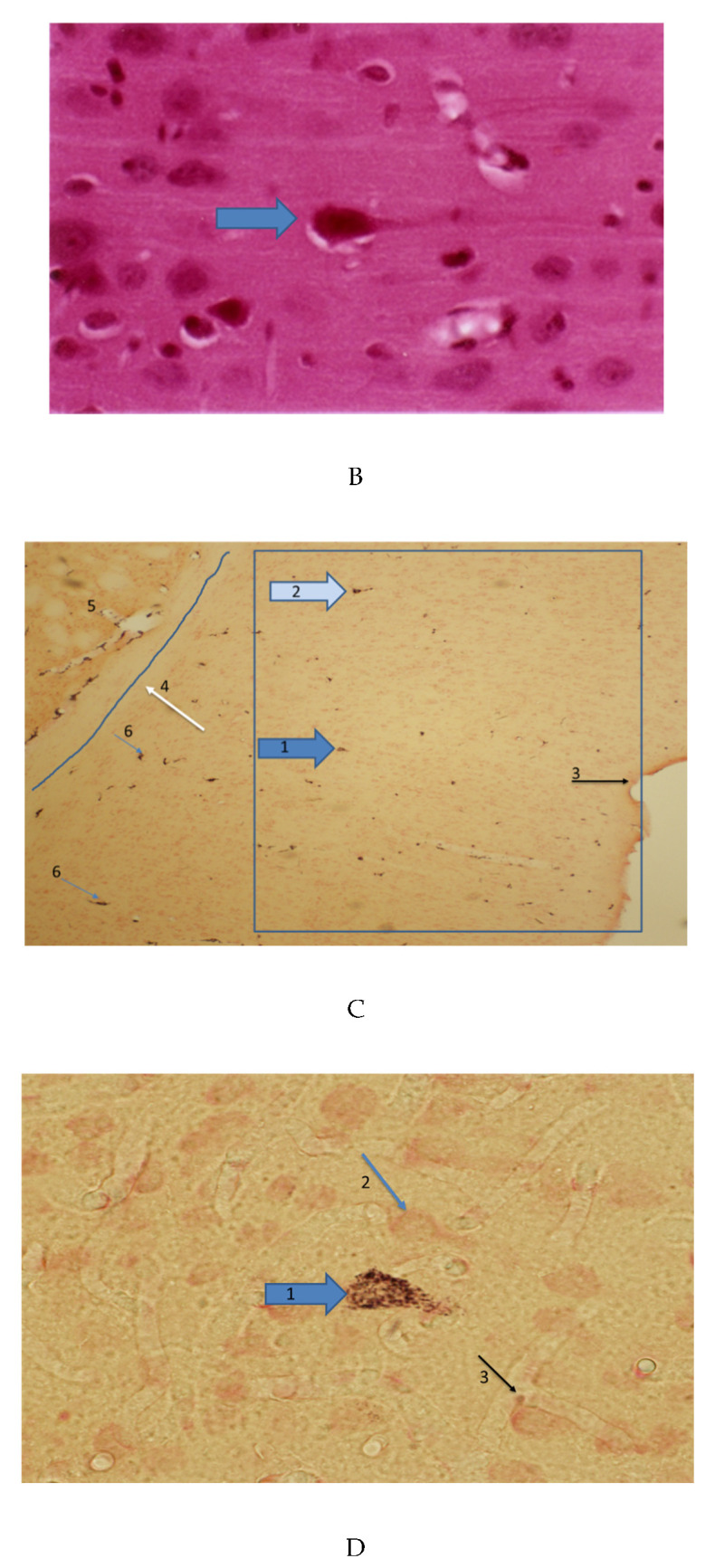

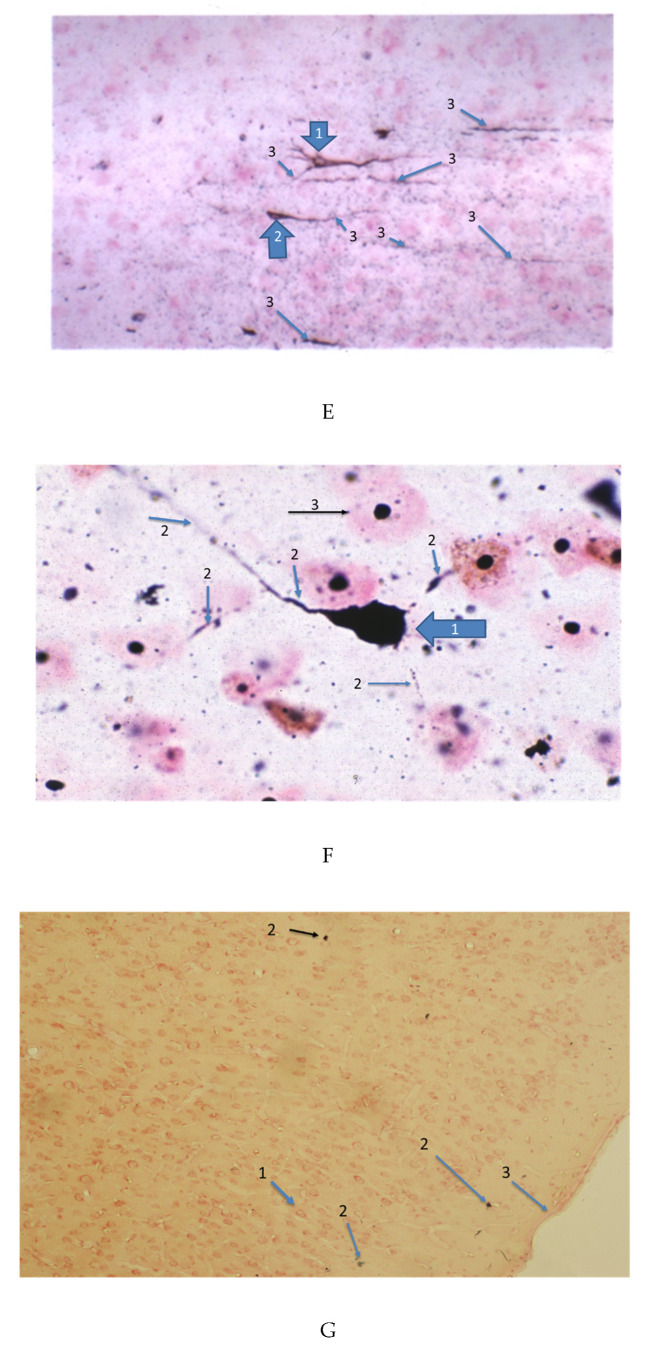

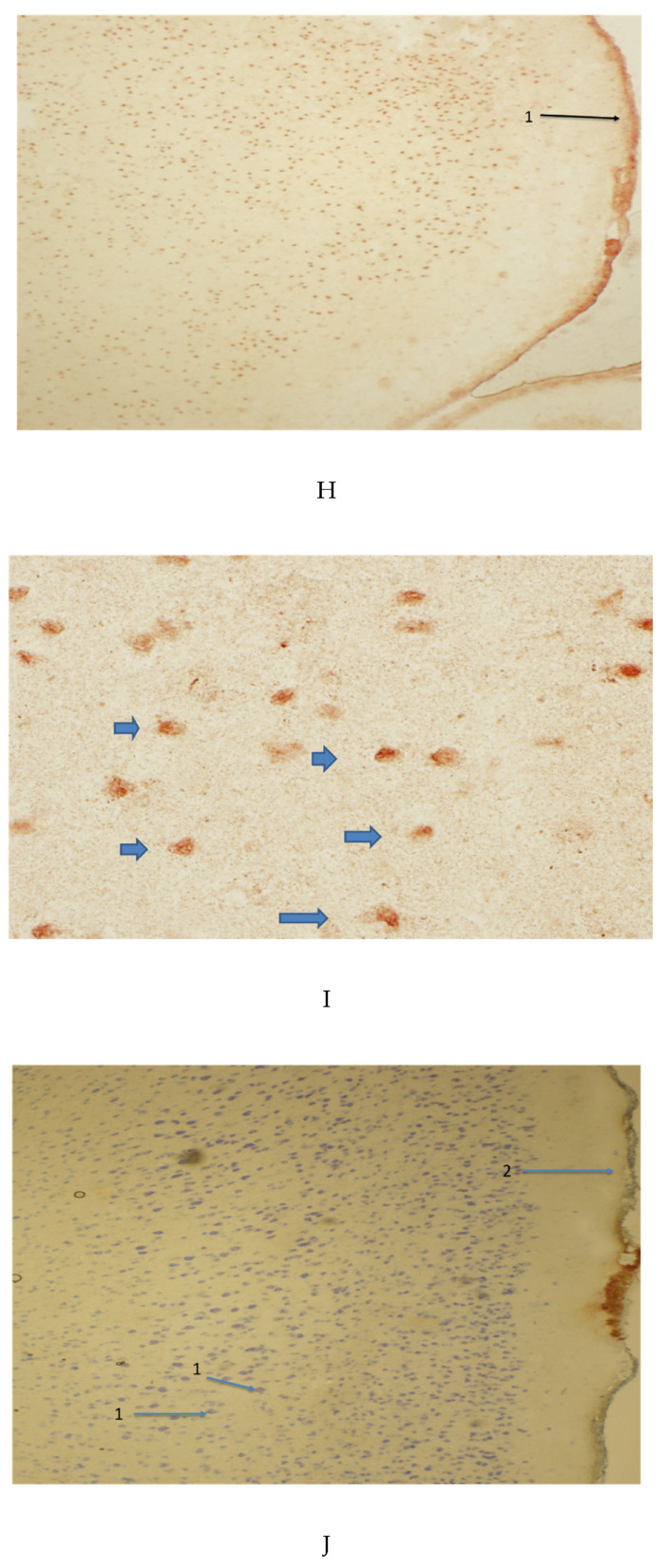

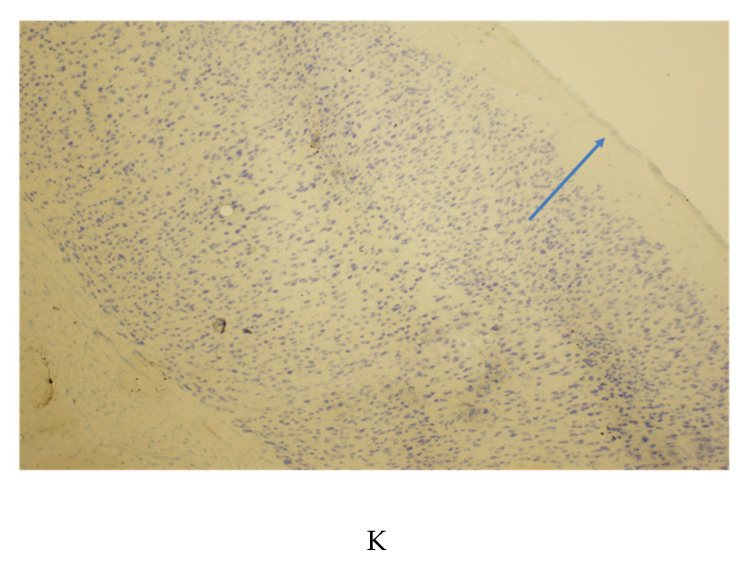
(**A**) HIV-1 transgenic rat (HIV-1TGR) coronal section of cortex stained with hematoxylin and eosin. This image of the 10-micron thick section was taken at 4×. In the present section, the surface of the brain is to the right (blue arrow), and the box encloses the cortical areas where both normal neurons (Arrow 3) and degenerating neurons (Arrow 4) could be found. Number 2 and the blue line it is pointing to is a white-matter tract beneath the cortex, and the area surrounding Number 1 is the subcortical gray matter. CXCR4-positive neurons can be found through the cortical area with immunohistochemistry. (**B**) HIV-1TGR coronal section of cortex stained with hematoxylin and eosin. This image of the 75-micron thick section was taken at 40×. H&E sections of the HIV-1 TG rat brains were analyzed for inflammation and other abnormalities. Inflammatory infiltrates were absent. A number of changes were noted, such as the “dead and red” neurons. Such neurons could be found at a very low frequency in the cortex. An arrow points to an example of a “dead and red” neuron. Neural cell death was confirmed with CSS. (**C**) The HIV-1TGR coronal section of the cortex is stained with CSS and counterstained with neutral red. This image of the 75-micron thick section was taken at 4×. In the present section, the surface of the brain is to the right (black Arrow 3), and the box encloses the cortical area where both normal neurons (stained red with no silver impregnation) and degenerating neurons (Arrows 1 and 2) could be found. Arrow 4 and the blue line it is pointing to locates the subcortical white-matter tract. The area surrounding Number 5 is the subcortical gray matter. Small blue arrows point to silver artifact (Arrow 6) precipitation and partial degenerating processes. Non-neural cells, such as microglia, may be labeled. A high-power image of a degenerating neuron (Number 1) can be found in Figure 1D. (**D**) The HIV-1TGR coronal section of the cortex is stained with CSS and counterstained with neutral red. This image of the 75-micron thick section was taken at 40×. This high-power image of the degenerating neuron from Figure 1C demonstrates pyramidal neuronal morphology. Degenerating neurons were often found in isolation. CSS at Arrow 1. Arrow 2 points to a normal fast red-staining cortical neuron. Arrow 3 points to a capillary. (**E**) The HIV-1TGR coronal section of the cortex is stained with CSS and counterstained with neutral red. This image of the 75-micron thick section was taken at 40×. This high-power image of the CSS demonstrates several degenerating neurons in close proximity to each other. The silver degeneration stain reveals that several cortical pyramidal cells are in different states of degeneration. Large arrows identify degenerating neurosomata of cortical pyramidal cells (Numbers 1 and 2). Smaller arrows point to degenerating neural processes (Arrows 3). (**F**) The HIV-1TGR coronal section of the cortex is stained with CSS and counterstained with neutral red. This image of the 75-micron thick section was taken at 60× water immersion. The degenerating neuron is darkly labeled (Arrow 1); its processes (Arrows 2) are granularly labeled, suggesting this neuron has been atypical for some time. Arrow 3 points to a normal neuron stained with neutral red. (**G**) Nontransgenic control rat coronal section of the cortex is stained with CSS and counterstained with neutral red. This image of the 75-micron thick section was taken at 4×. Brains of nontransgenic control rats did not display any observable degenerating neurons. Artifactual silver precipitate could be identified (Arrows 2) throughout the sections. Red blood cells and capillaries could display silver precipitate, but no morphologic neurons could be identified with silver impregnation. Arrow 1 points to an example of a neutral red-staining cortical neuron. Arrow 3 points to the brain surface. (**H**) The HIV-1TGR coronal section of the cortex is stained by CXCR4 immunocytochemistry. DAB (3,3’-diaminobenzidine) was used as a chromogen. This image of the 10-micron thick section was taken at 4×. The low power image demonstrates many positive cortical neurons and glia cells (brown reaction product). Arrow 1 points to the surface of the brain. (**I**) The HIV-1TGR coronal section of the cortex is stained by CXCR4 immunocytochemistry. DAB was used as a chromogen. This image of the 10-micron thick section was taken at 40×. Immunostaining to CXCR4 revealed many positive cells of differing morphology. Arrows point to a number of DAB-labeled cells. (**J**) Negative control for CXCR4 immunocytochemistry in the cortex of the HIV-1TGR. Original magnification 4×. As there was a lack of specific staining, hematoxylin was used to demonstrate the presence of cortical cells. Arrow 1 points to cortical neurons, and Arrow 2 points to the surface of the brain. (**K**) Negative control for CXCR4 immunocytochemistry in the cortex of the nontransgenic rat. Original magnification 4×. As there was a lack of specific staining, hematoxylin was used to demonstrate the presence of cortical cells. Arrow 1 points to the surface of the brain.

The cortex shows signs of neuronal degeneration. An H&E stain at low power (Figure 1A) demonstrates the orientation of the cortex. The surface of the brain is to the right, and the underlying basal ganglia are to the left. It shows increased eosinophilia in a few cells, suggestive of dying neurons. At higher power (Figure 1B), this effect can be noted. CSS reveals that cortical pyramidal cells were degenerating (Figure 1C). Their morphology is clearly delineated by the silver stain (arrows 1 and 2). This can be seen at higher power in Figure 1D (Arrow 1). Neurons in varying states of degeneration can be identified by the differing degrees of silver impregnation, as seen in Figure 1E,F. Dark solid impregnation suggests a recently dead neuron, while one with a granular appearance suggests it died at an earlier time point. Inflammatory infiltrates surrounding such cells were not observed. Nontransgenic control rats were also stained with CSS. No evidence of neuronal impregnation with silver was noted in these controls (Figure 1G). 

The CXCR4 antibody labeled many cells throughout the cortex, and each cortical layer included pyramidal cells [55]. The CXCR4-labeling of the cortex at low power is shown in Figure 1H, while a high-power image is found in Figure 1I. The omission of the primary antibody for negative control abolished staining in both the HIV-1 TGR and the nontransgenic control (Figure 1J,K).

### 3.2. Hippocampal Pathology

**Figure 2:**
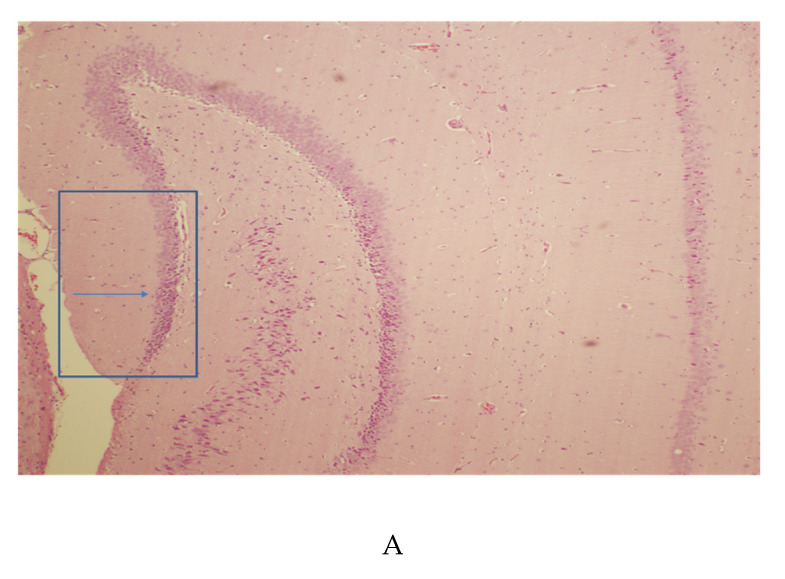

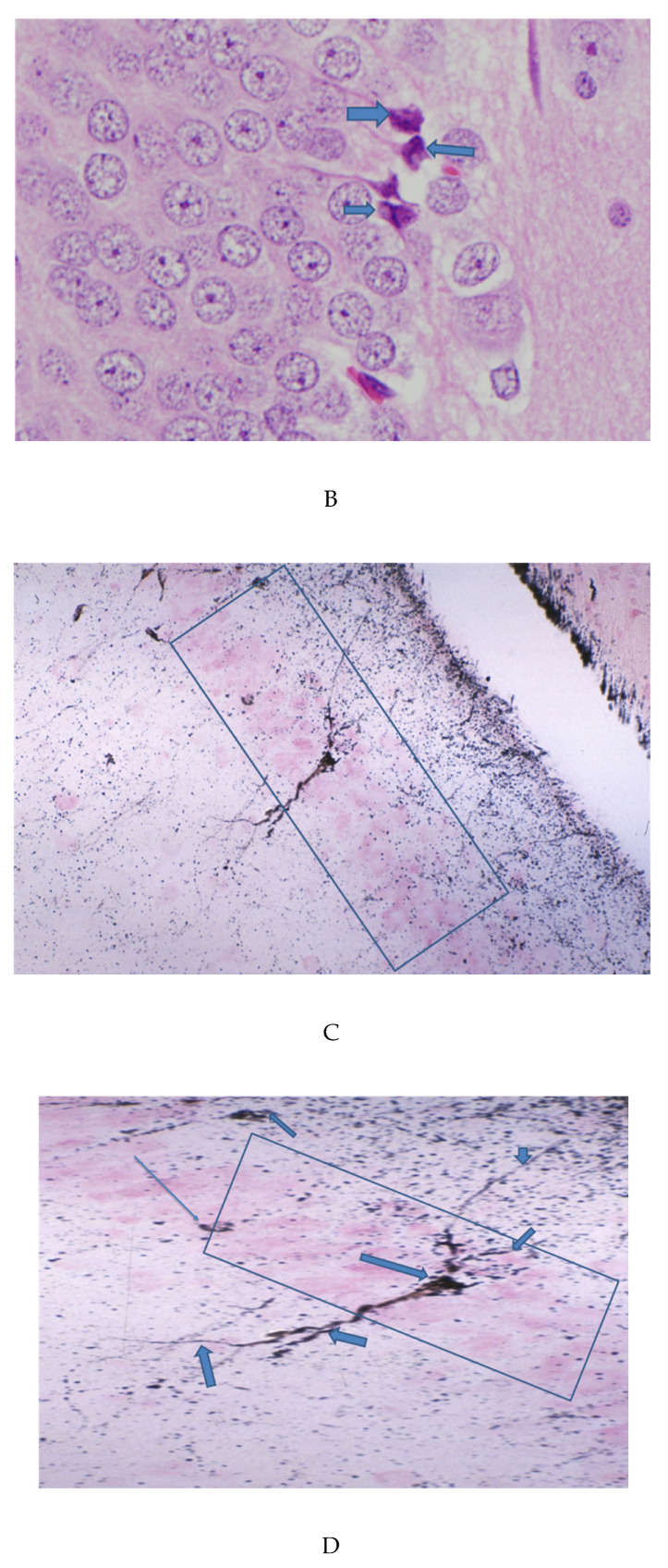

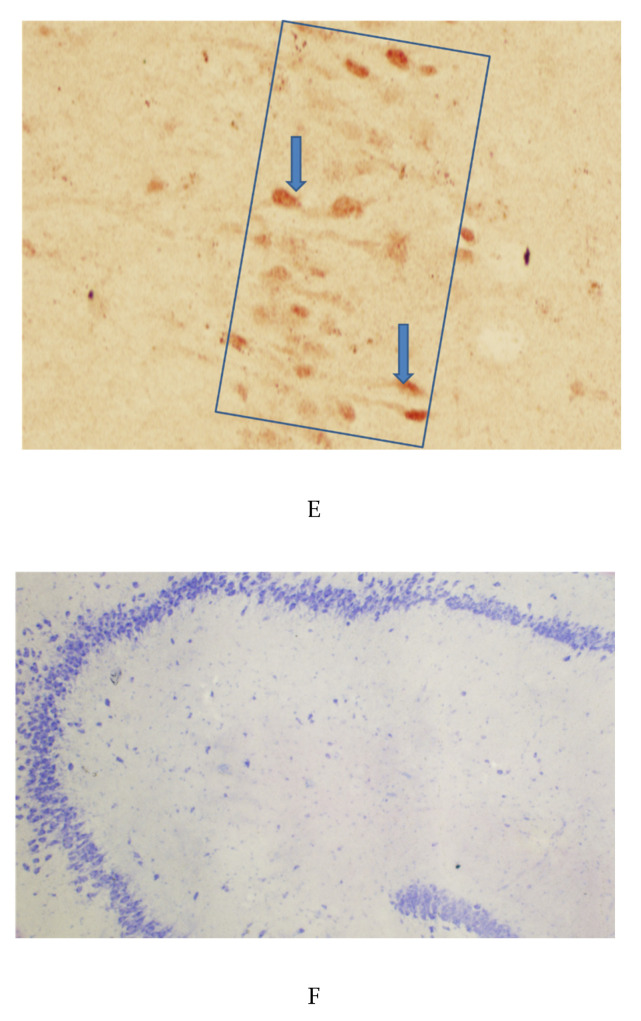
(**A**) HIV-1TGR coronal section of the hippocampus stained with hematoxylin and eosin. This image of the 10-micron thick section was taken at 4×. In the present section, the box encloses the hippocampal areas where both normal neurons and degenerating neurons could be found. The Blue arrow points to the granular cell layer where degenerating neurons could be found. See also Figure 2C. (**B**) HIV-1TGR coronal section of the hippocampus stained with hematoxylin and eosin. This image of the 10-micron thick section was taken at 60×. The H&E stain shows signs of nuclear atypia, consistent with neuronal degeneration. The degenerating neurons were very infrequent, but they could be found in different areas of the hippocampus, like CA3 and the dentate gyrus. Arrows point to several examples. This degeneration was confirmed with CSD (see also Figure 2C). (**C**) The HIV-1TGR coronal section of the hippocampus is stained with cupric silver stains (CSS) and counterstained with neutral red. This image of the 75-micron thick section was taken at 20×. The box encloses the area of granular neurons, which appear lightly stained with neutral red. One hippocampal neuron is labeled with the CS stain and reveals its hippocampal morphology. High power further demonstrates the morphology in Figure 2D. (**D**) HIV-1 TGR, original magnification 60× CSS with neutral red counterstain. Degenerating neurons in CA3 could be identified in the granular layer. The box outlines the dentate gyrus granular cell layer, and the arrows point to degenerating cells and their processes. (**E**) Immunocytochemistry of CXCR4 in the HIV-TGR hippocampus. Original magnification 40×. DAB brown chromogen was used. Positive-staining neurons could be found throughout the hippocampus. The box shows the area of the dentate granular cells. It can be seen that many of this cell type was labeled for CXCR4. Arrows point to several examples. (**F**) Negative control for CXCR4 immunocytochemistry in the hippocampus of the HIV-1 TG rat. Original magnification 4×. As there was a lack of specific staining, hematoxylin was used to demonstrate the presence of the cells. Nonspecific staining was not detected with the omission of the immune sera.

The hippocampus showed signs of individual neuronal degeneration. A low power H&E provides an orientation of the hippocampus (Figure 2A). The blue box shows a portion of the granular cell layer in which degenerating neurons could be found. At high power, individual neurons display degenerative changes and nuclear atypia (Figure 2B). No signs of inflammatory infiltrates were found. The silver stain confirmed neuronal degeneration (Figure 2C), and a higher power image (Figure 2D) demonstrates the morphology of the hippocampal granular neuron, together with its degenerating terminals. When the silver impregnation filled the neuron (Figure 2D), the morphology indicates it is a hippocampal cell surrounded by normal hippocampal neurons, revealed with the counterstain. Normal hippocampal granular cells, which were only stained with neutral red (Figure 2C,D), are found within the blue box, together with a degenerating one. Examples of two other degenerating neurons that are only partially in the section can be found to the left and above the central staining one (see arrows). 

In Figure 2E, CXCR4-labeled hippocampal cells are evident by the brown reaction product of DAB. Hippocampal neurons lying within the dentate granular layer are shown in the box. It can be noted that most of these cells are labeled with CXCR4. Some labeled cells display partial neuronal processes. The negative control of the hippocampus did not reveal any labeling. It was counterstained with hematoxylin to reveal the granular cells (Figure 2F).

### 3.3. Striatal Pathology

**Figure 3:**
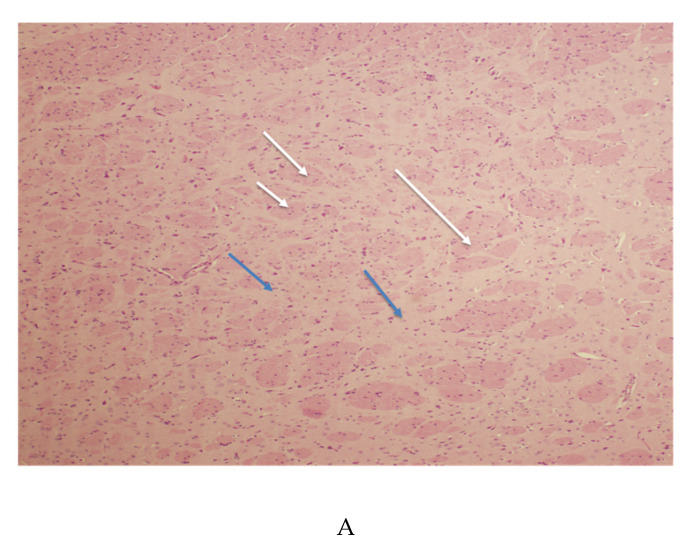

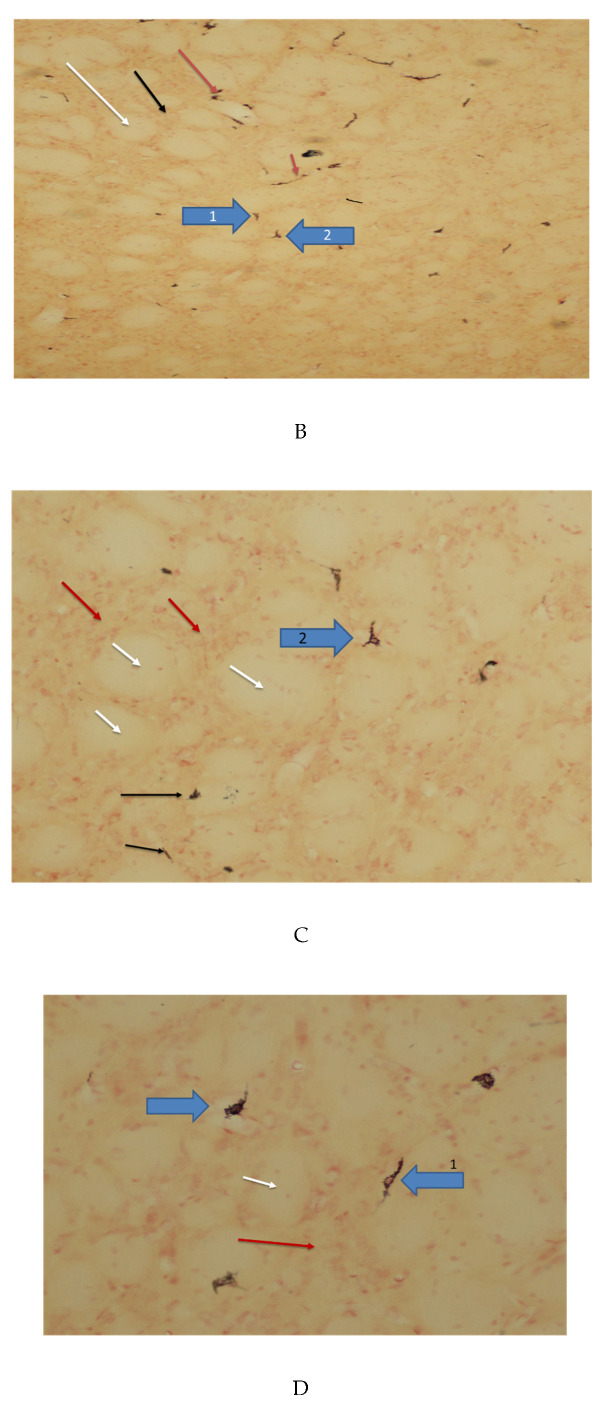

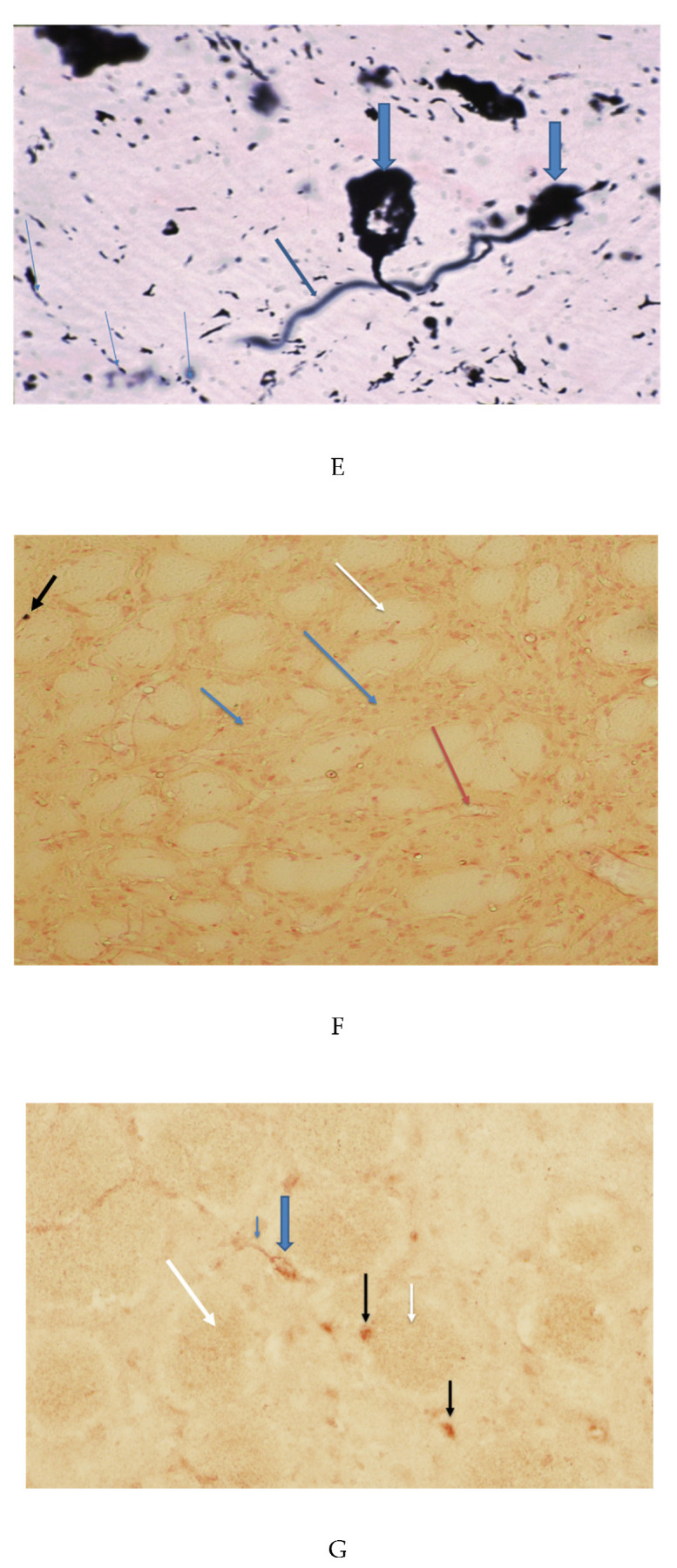

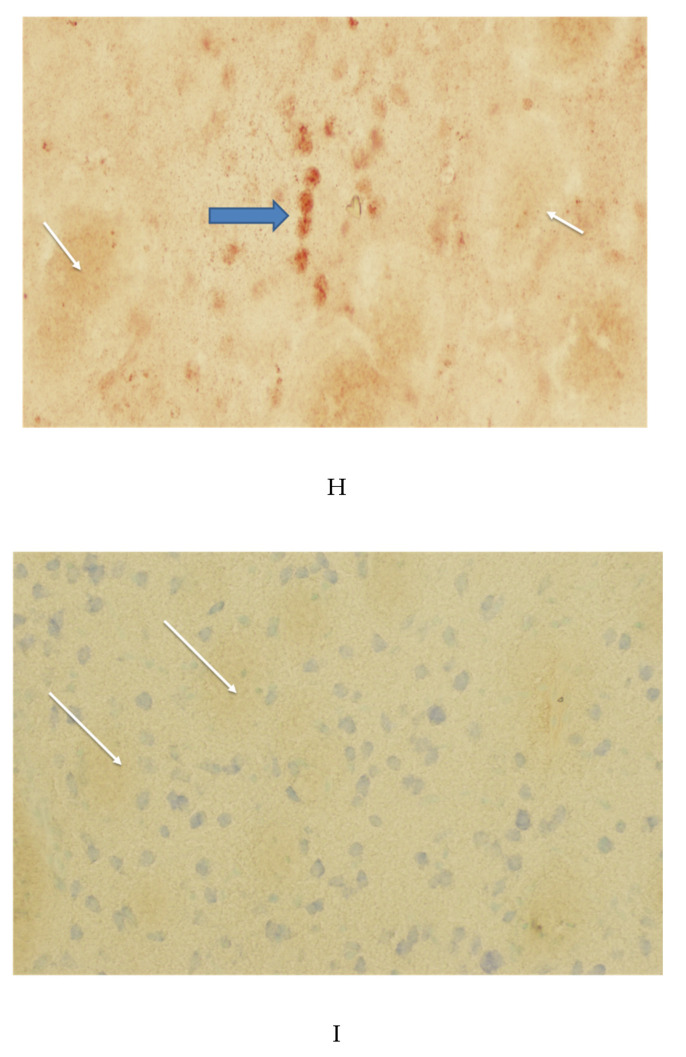
(**A**) HIV-1TGR coronal section of the caudate/putamen stained with hematoxylin and eosin. This image of the 10-micron thick section was taken at 4×. The blue arrows are pointing to gray matter areas. The white arrows are pointing at the fibers of passage. (**B**) HIV-1TGR coronal section of the caudate/putamen stained with CSS. This image of the 75-micron thick section was taken at 10×. The blue arrows are pointing to degenerating neurons. The white arrows are pointing at the fibers of passage. The black arrow points to a gray matter area. The red arrow points to an infiltrated capillary. See Figure 3C for a higher-powered image of the cell at Arrow 2. (**C**) HIV-1TGR coronal section of the caudate/putamen stained with CSS. This image of the 75-micron thick section was taken at an original magnification of 20×. The blue arrow (Arrow 2) is pointing to the degenerating neuron from Figure 3B. The white arrows are pointing at the fibers of passage. The red arrows point to a gray matter area. The black arrows are pointing to silver precipitate. (**D**) HIV-1TGR coronal section of the caudate/putamen stained with CSS. This image of the 75-micron thick section was taken at 20×. The blue arrows are pointing to the degenerating neuron. The white arrows are pointing at the fibers of passage. The red arrows point to a gray matter area. (**E**) HIV-1TGR coronal section of the caudate/putamen stained with CSS and counterstained with neutral red. This image of the 75-micron thick section was taken at 60×. The blue arrows are pointing to the degenerating neuron. The neuron is of a small or moderate type. In addition, it is possible to identify the degenerating fibers. Large arrows point to the degenerating neurosomata, while small arrows point to degenerating fibers. (**F**) A coronal section of the caudate/putamen from the nontransgenic control rat stained with CSS. It was also counterstained with neutral red. This image of the 75-micron thick section was taken at an original magnification of 20×. The blue arrows are pointing at the gray matter (cells stained red). The white arrows are pointed at the fibers of passage. The black arrow is pointing to silver precipitate. No degenerating neurons or other cell types were noted. (**G**) HIV-1TGR coronal section of the caudate/putamen stained with CXCR4 immunohistochemistry. Original magnification 40×. DAB brown chromogen was used. Positive CXCR4-labeled cells could be found throughout the striatum. The CXCR4-labeled cells displayed several morphologies in the striatum. One cell type appears to be microglia-shaped. The large blue arrow points to the elongated cell body, and the small arrow points to the process. The white arrows are pointing to the white matter tracts and the black arrow to other positive cells. (**H**) HIV-1TGR coronal section of the caudate/putamen stained with CXCR4 immunohistochemistry. Original magnification 40×. DAB brown chromogen was used. Positive CXCR4-labeled cells could be found throughout the striatum. The CXCR4-labeled cells displayed several morphologies in the striatum. The large blue arrow points to the more rounded cell types. The white arrows are pointing to the white matter tracts. (**I**) HIV-1TGR coronal section of caudate/putamen negative control immunohistochemistry. Original magnification 10×. DAB brown chromogen was used. Hematoxylin counterstain reveals the cells. No labeled cells could be found in the striatum when the primary antibody was substituted with the nonimmune serum. White arrows point to the fibers of passage.

Low magnification of a hematoxylin and eosin stain of the caudate/putamen reveal the fibers of passage (white arrows) and the cells (blue arrows) that make up this structure (Figure 3A). Areas in the caudate/putamen, which displayed degenerating neurons, are observed in Figure 3B. In this low-power magnification, it is possible to see the structure of the striatum, which includes the fibers of passage and cells stained with neutral red. Several cells labeled with CSS can be found. Arrows 1 and 2 point to the degenerating cells. Figure 3C shows their morphology at higher magnification. Figure 3D shows cells of differing morphology labeled with CSS. The morphology of one cell is suggestive of microglia. In Figure 3E, a high-power image displays a neuron with its process. This appears to be an interneuron. In addition, it is also possible to observe the degenerating neuronal processes of that cell. These degenerating cells appear consistent with neuronal morphology in the caudate [56]. Nontransgenic control rats were also stained with CSS. No evidence of neuronal impregnation with silver was noted in the caudate/putamen of these animals (Figure 3F). 

The cell types expressing CXCR4 were demonstrated by immunohistochemistry. They appear to display a more diverse morphology. Figure 3G shows microglia-like morphology, while Figure 3H shows morphology consistent with astrocytes or small neurons. The negative control of the caudate did not reveal any labeling. It was counterstained with hematoxylin to reveal the cells (Figure 3I).

### 3.4. Immunohistochemical Identification of gp120 and Tat in the Brain 

The brain was stained with both gp120 and Tat. This produced a negative result so far, as with positive neurons or neuronal cells. Only the rare lymphocyte-appearing cells seem to be stained. At the level of immunohistochemical staining, cells in the nervous system were not positive. To further confirm the effectiveness of the immunohistochemical approach, we labeled gp120 and Tat in the spleen. This produced positive results, consistent with our earlier findings. 

### 3.5. Spleen Pathology

#### 3.5.1. Immunohistochemical Identification of gp120 and Tat

**Figure 4:**
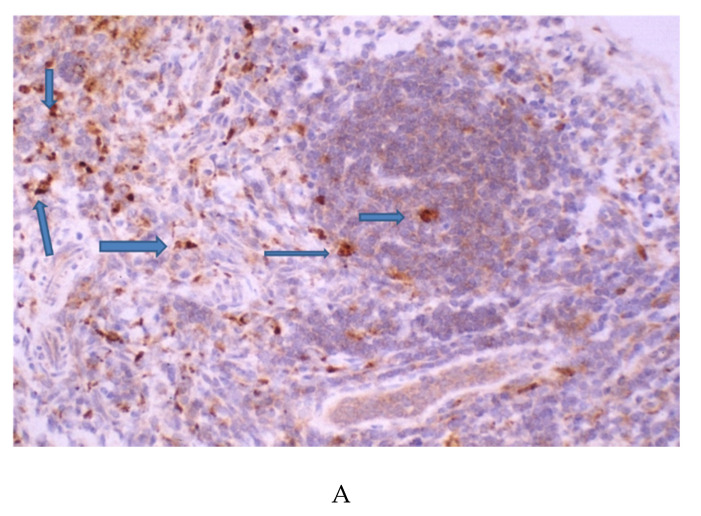

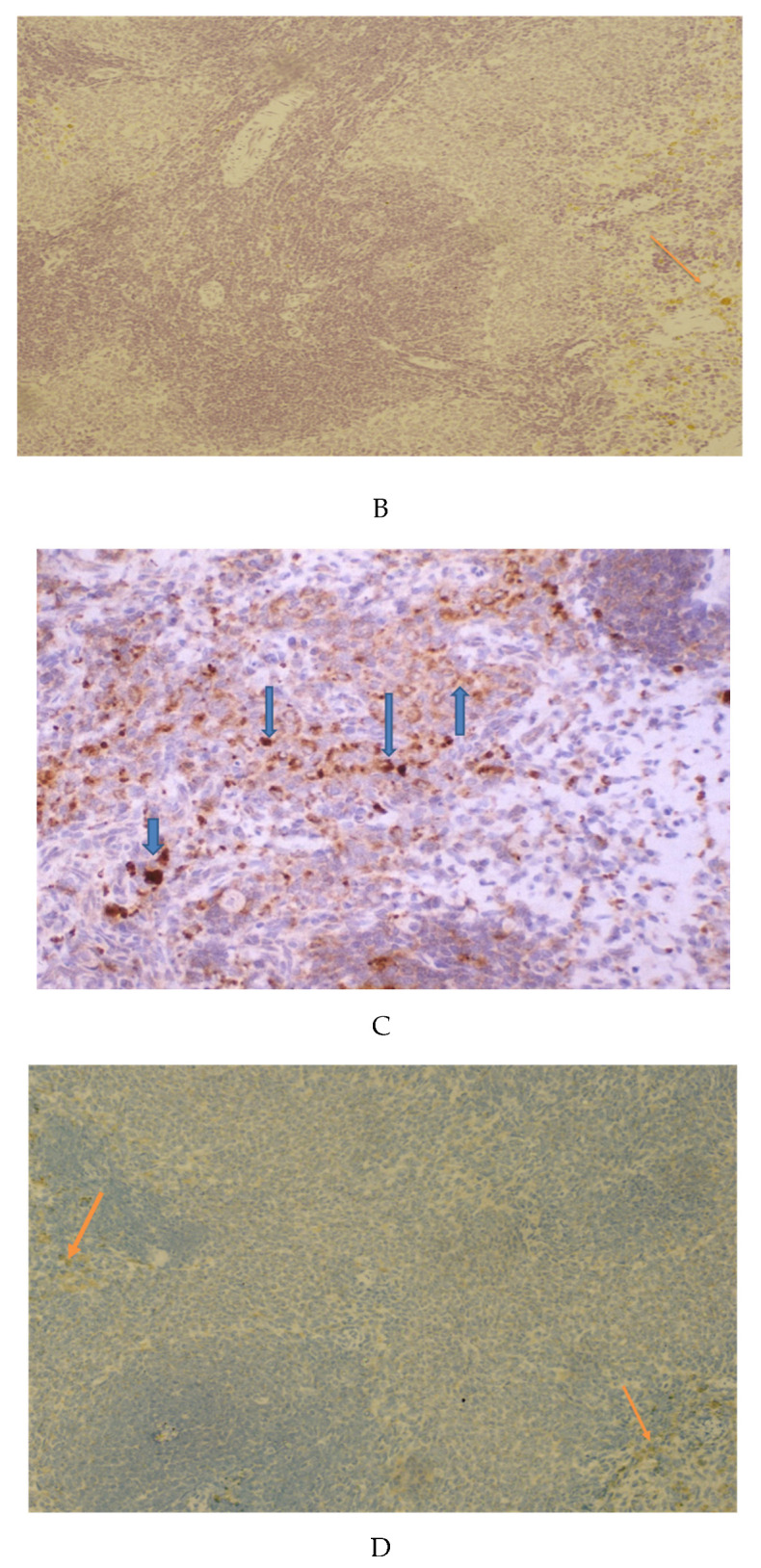

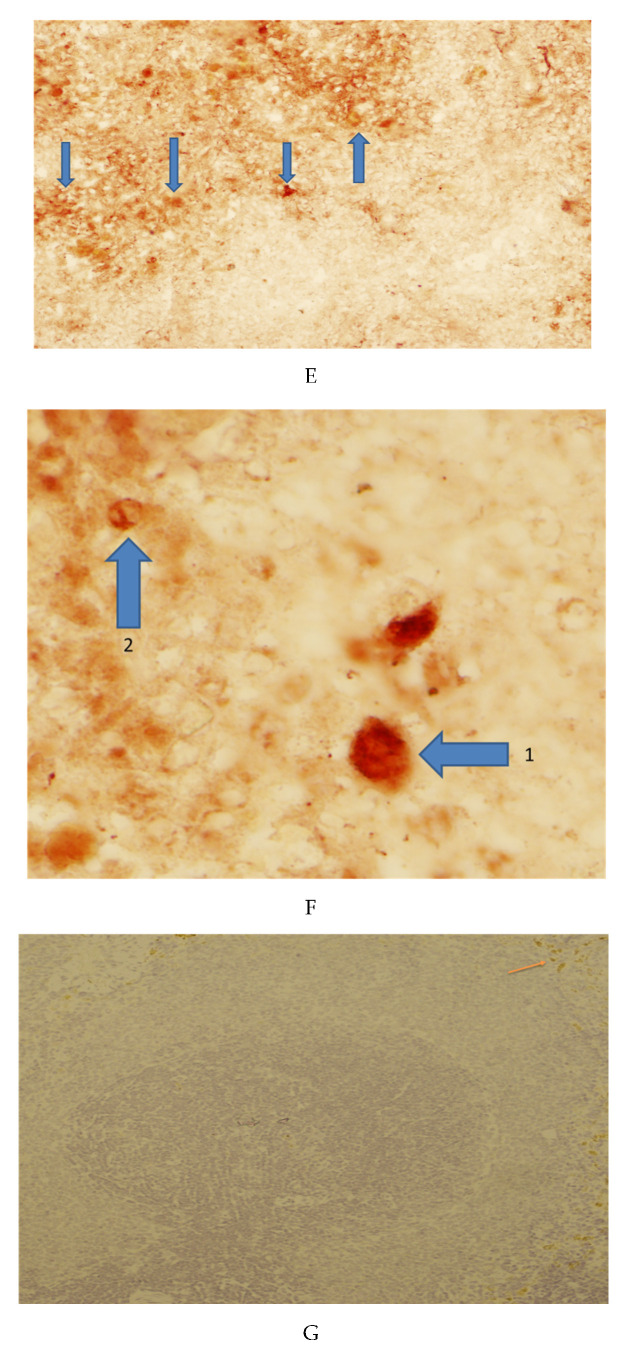
(**A**). Transgenic rat (TGR) spleen, original magnification 40×. gp120 immunostaining with brown DAB chromogen and counterstain with hematoxylin. Blue arrows point to several gp120-stained cells. (**B**) HIV-1TGR spleen, original magnification 10×. Negative control for gp120 immunostaining. Brown DAB chromogen and counterstain with hematoxylin. Orange arrow points to hemosiderin, not a DAB label. (**C**) TAT immunostaining of the HIV-1TGR spleen. Original magnification 40×. Brown DAB chromogen and counterstain with hematoxylin. Blue arrow points to several examples. (**D**) HIV-1TGR spleen, original magnification 10×. Negative control for TAT immunostaining. Brown DAB chromogen and counterstain with hematoxylin. Orange arrow points to hemosiderin, not a DAB label. (**E**) CXCR4-staining of the HIV-1TGR spleen. Original magnification 20×, DAB chromogen. Positively labeled cells can be found throughout the spleen. The blue arrows point to examples of CXCR4 positive labeled cells. (**F**) CXCR4 staining of the TGR spleen. Original magnification 60×, DAB chromogen. Positively labeled cells can be found throughout the spleen. There are several different cell types labeled based on size. Arrow 1 points to an example of a large cell, while Arrow 2 points to a smaller cell type. (**G**) HIV-1TGR spleen, original magnification 10×. Negative control for CXCR4 immunostaining. Brown DAB chromogen and counterstain with hematoxylin. Orange arrow points to hemosiderin, not a DAB label. No nonspecific labeling was identified.

The spleen displayed numerous labeled gp120-positive (Figure 4A) and Tat-positive (Figure 4C) cells. Examination of the spleen sections reveals that the labeled cells can be found in both the red and white pulp areas. The histopathology found in the spleen was consistent with previous reports. At times, the ratio of red pulp to white pulp appeared altered, consistent with the attrition of splenocytes [43]. Negative controls for gp120 (Figure 4B) and Tat (Figure 4D) were negative.

#### 3.5.2. Identification of CXCR4 in the Spleen

CXCR4-labeled cells were found throughout the spleen in the low-power image of Figure 4E. The CXCR4-antibody-labeled splenocytes are of different sizes. Cells of a larger type are suggestive of macrophages and show marked staining in the high-power image in Figure 4F. The spleen in the HIV-1TGR has been reported to display marked apoptosis and cell loss [43]. The controls for CXCR4 were appropriate for the stains used. The CXCR4 labeling was not observed when the primary antisera were replaced with nonimmune sera (Figure 4G).

## 4. Discussion

In view of the changing characteristics of the HIV disease, it is necessary to develop model systems to address the emerging types of HIV pathology. Due to the ability of the HIV-1TGR to recapitulate HIV disease in several organs, we propose the HIV-1TGR as a model for the analysis of HIV noninfectious comorbidities. To this end, we noted that HIV receptor presence in the rat may provide an explanation of the specific organ pathology that has been observed in this model. While upward of 100 papers have been published using this model, an explanation for the specific cell pathology has been lacking. Analysis of the rat CXCR4 receptor and cell culture experiments on viral infectivity have demonstrated that rat CXCR4 can bind to gp120, with subsequent cell dysfunction. 

Previous research reports that two important HIV proteins (Tat and gp120) are expressed in this animal [43,46,57] and are available to the brain in sera, CSF, or by specific local cell expression, which could be part of an important pathologic mechanism. We, therefore, examined the possibility of gp120 and Tat expression in the HIV-1TGR brain. As gp120- and Tat-positive cells are very rare in the brain, this supports the view that systemic availability of at least gp120 may be the contributing mechanism. If cells in the brain express the viral receptor CXCR4, this would make such a mechanism feasible. We then demonstrated the presence of gp120 receptor CXCR4 in HIV-1TGR tissues. 

The tissues selected were the brain and spleen, as research has revealed important NICM pathology in both these organs. In the HIV-1TGR, degenerating neurons were found in the pyramidal cells of the cortex and the granular cells of the hippocampus, microglia and interneurons in the caudate/putamen. These areas of the brain have been implicated in the AIDS dementia complex. The finding of degenerating neurons in the striatum is also important for their relationship to the dopaminergic system. Destruction of these neurons can contribute to motor problems. Degeneration of hippocampal neurons can contribute to memory problems. It was also noted that there were cells of a microglia morphology. If gp120 binds to these cells, the possibility of free radical production may be stimulated. The mechanism by which these cells of the brain or other tissues are dying is, however, poorly understood; therefore, demonstrating the expression of CXCR4 would provide one such mechanism. 

CXCR4 labeling was found in the pyramidal cells of the cortex and the granular cells of the hippocampus, microglia, and interneurons in the caudate/putamen. These cell types were also found to be undergoing degeneration, as demonstrated by CSS. The expression of CXCR4 in these cells suggests a reason for why these particular cells are dying. CXCR4 labeled many cells in the spleen. The spleen has previously been reported to display marked apoptosis. The mechanism of splenic cell death may be more complex. The spleen has cells that produce gp120, Tat, and Nef [43]. In addition, HIV proteins like Tat can increase the expression of CXCR4 [58]. How this relates to the production of pathology in the spleen will require further study. The splenic microenvironment is complex due to the expression of multiple transgenes. 

The brain, on the other hand, did not present with many cells displaying HIV transgene products at the level of immunocytochemical sensitivity. While gp120 has been found in sera and CSF, the level at any given time may be low. The process, however, is chronic, and cell dysfunction and death develop over an extended time period. These observations are consistent with a receptor-mediated pathologic mechanism. It also contributes an explanation for the observation of selective neuronal vulnerability and why it takes so long for the symptoms to present in both this model and in the clinical condition. 

HIV-1TGR parallels human pathology in particular due to CXCR4 receptor distribution in the tissues. If chronic exposure to gp120 leads to the increased destruction of CXCR4-positive cell types, then those tissue/organs will display increasing dysfunction. CXCR4 presence provides a link between the virus (in humans and the transgene product in the HIV-1TGR) and the cellular mechanisms leading to that dysfunction. Knowing which cells are participating in the pathology can lead to more focused molecular analysis. For studying the HIV dementia complex, there is now a pathway that can be followed from behavior to the neuropathology of the specific neuronal cell type to neurologic dysfunction. This can provide research avenues for both cellular and molecular approaches to study the disease process. 

## 5. Conclusions

In view of the supportive literature and our current observations, the HIV-1TGR is a unique model for the study of HIV noninfectious comorbidities, and our work describes in detail some of the unique advantages of such a model. In addition, it provides several insights for experimental intervention to prevent cell death or dysfunction in the HIV disease. Some therapeutic approaches are also possible. The first is the inhibition of HIV transcription, the next is regulation of immune modulation, and the third is CXCR4 receptor blockage. As each of these factors contributes to HIV disease in general and to the development of NICMs, the HIV-1TGR model offers the possibility of investigating the NICM disease process at several levels.
